# The Fan System of Heating, Ventilating and Air Supply of Certain Buffalo Schools

**Published:** 1901-08

**Authors:** Henry Reed Hopkins

**Affiliations:** Buffalo, N.Y.


### BUFFALO MEDICAL JOURNAL.

VOL. XLI.—LVII. AUGUST, 1901. No. 1.

### ORIGINAL COMMUNICATIONS.

### The Fan System of Heating, Ventilating and Air Supply of Certain Buffalo Schools.

By HENRY REED HOPKINS, M. D., Buffalo, N.Y.

THE public schools of Buffalo present to the student of hygiene many interesting topics for consideration. This is true with greater particularity of the systems of heating and ventilation in use, for here we may observe instances of the gravity, the vacuum, the plenum systems, and in many instances combinations of two of these systems. Here we may observe that the well known principle, that where there are numerous and diverse treatments in use for a given disease, the ideal treatment has yet to be discovered, seems to apply to the problem of school ventilation.

In this important subject, touching the health and future usefulness of thousands of our children, during the more susceptible period of their lives, the law of the survival of the fittest, has yet to pick the winner, the ideal system of heating, ventilating, and air supply. The system having at once the highest marks in economy, simplicity, adaptability and efficiency, has yet to come, and to show that it has come by displacing all others.

The fan system of ventilation rests upon the well known fact that air is a fluid which, with the use of the proper mechanical device, may be lifted as water is lifted from the bottom of the well; or blown through an air shaft, as water is forced through a hose to put out a fire, or to sprinkle the flowers upon the lawn. The fan system is found at work after these two orders—the vacuum, the fan in the attic, lifting the foul air from the rooms below, and the plenum—the fan in the basement, forcing air into the rooms above, heating these rooms, displacing their foul air by an equal volume of warm, pure air, thereby keeping up a constant state of perfect ventilation and air supply.

The vacuum system, the fan in the attic, is at once the simpler and more modest in its undertaking, aims only to empty the rooms of foul air, and assumes no responsibility for heating and air supply; has a single purpose—ventilation. Its mechanism includes a revolving fan in an air chamber in the attic. This air chamber is connected on one side by a system of foul air shafts with the several rooms of the building, and on the other side opens through the roof to the outer air. The work of the fan is to raise the air from the rooms below and drive it out of doors through the roof.

The plenum system, the fan in the basement is more ambitious of purpose and correspondingly complex and complicated of mechanism. It assumes the responsibility of heating, ventilating, and air supply; a matter of comfort, a question of health, and the necessity of life. The fan in the basement is exposed on one side to the outer air, and choice may be exercised as to the source of air, it may come from a hole in the ground or it may be taken from the bottom of a tall air shaft, the top of which is above the line of ground air or dust contamination. However taken, when once taken the plenum system assumes entire control of the air supply for the building.

All the air for all the occupants is seized, directed, screened from dust, warmed, and when required moistened and distributed according to their several necessities to the different rooms; equally diffused to all parts of these rooms giving in exact and generous allowance, warmth, ventilation, and air supply to each and every occupant; and having done all this, still under the direction and control of the fan is gathered by the various outlets for that purpose made and provided and driven up through the foul air shafts, through the several stories, through the attic, through the roof to the open air beyond.

More than thirty Buffalo schools are fitted with this plenum fan system. Some of these have been in use for more than ten years, and the cost of the apparatus is near $250,000.

It is the purpose of this paper to inquire how this system is performing—what part of its undertaking is being fulfilled.

Since 1896 the Buffalo School Association, through its visiting committee, a committee including many of our most enthusiastic and enterprising medical men, has been engaged in studying the sanitary condition of our schools. The reports of this association called the attention of the public to the fact, that in a large majority of our schools the ventilation was most unsatisfactory.

The Board of School Examiners also took up the investigation and soon reached the conclusion quite in harmony with that of the School Association,—an interesting and singular fact, inasmuch as the pioneer body is a self-formed and self-invited body, and the former is regularly appointed and salaried and withal charged with just such responsibility. As a result of this agitation against the nonventilated state of the class-rooms of our schools, the health department ordered that an examination into the facts of the case be made by our city chemist. Such examination was made in the winters of 1900 and 1901, and the reports are now on file in the health department. From these reports the facts of this paper are extracted.

Before taking up the pertinent facts of these reports, it seems worth observation that the first information which the city had as to the working of its school ventilation, was the reports of the city chemist, made by the order of the health department. Some $250,000 had been invested in a most complicated and artificial mechanism, which presumed to heat, ventilate and keep supplied with breathing air, the class rooms of more than thirty of our public schools, and yet there was not on file in any of our public offices, a record of a test of this mechanism, whereby proof was made as to how the important work was being performed. Contracts contained specifications relating to the nature and amount of work the mechanism was expected to perform, but tests and records of tests, showing how near the work approached the specifications were not to be found. The significance of this observation rests upon the fact that to those engaged in school work, teachers and pupils alike, heating is a matter of comfort, ventilation essential to health, and air supply the prime necessity of life—and that the mechanism in question presumed to furnish all of these.

During the winter of 1900, the city chemist examined and reported upon the air of the class rooms of schools Nos. 1, 12, 18, 37, 40, 56 and 57, all of which have the fan system, the fan being in the basement. In these examinations measurements and observations and tests of various kinds were made, all looking in the direction of testing the efficiency of the ventilation and air supply. Of these tests the more crucial is the observation of the amount of carbon dioxide present in the air of the class rooms, inasmuch as this respiratory product may safely be taken as the measure of the air borne excrement, the organic matter of human origin, the poison of rooms of human occupancy, to be rid of which ventilation is demanded.

The following are the amounts of carbon dioxide found in 10,000 volumes of air of the different rooms of the several schools: School No. 1, lowest, 7.2, the highest, 28.2—average of school, 18.53. No. 12, lowest, 23.9; highest, 47—average of school, 30.86. No. 18, lowest, 12.6; highest, 21.5—average of school, 16. No. 37, lowest, 17.3; highest, 44—average of school 27.23. No. 40, lowest, 6.4, highest, 10.7—average of school, 8.65. No. 56, lowest, 5.4, highest, 11.2—average of school 9.4. No. 57, lowest, 4.6, highest, 9.99—average of school, 8.20. Average for this group of seven schools, 16.98.

There were examined in these seven schools, forty-two rooms and of these rooms, two only were found to be ventilated and forty not ventilated. This is upon the standard of six parts of carbon dioxide in 10,000 volumes of air, as the maximum limit of permissible impurity.

In the winter of 1901, the city chemist examined the air of the following schools, Nos. 1, 12, 16, 18, 19, 37, 45 and 56. These with the work of the previous winter, as given above make a total of 196 rooms examined, of which number 31 were found to be ventilated and 165 were found not ventilated. All of these schools have the fan system of ventilation and all save No. 45 have the so-called plenum system—the fan in the basement.

If this work of the city chemist had done no more than to demonstrate the degree of nonventilation of the schools examined, it would even then have more than justified the judgment of the health department in ordering the work.

Fortunately the facts revealed by the examination of the winter of 1900 did but increase the desire of the health department for still further knowledge as to the atmospheric conditions of the school rooms; as to the rate of performance of the various parts of the mechanism for ventilation and air supply.

For instance, in his first winter examinations the city chemist had been content to measure the amount of air delivered to the various class rooms at the air inlets, but made no observations as to the work done by the ventilating apparatus proper, the outlets and their related foul air shafts. The blank directed by the health department for the work of 1901, contained columns for measurements of air movements at both inlets and outlets and the facts thereby determined seem of considerable importance, and possibly throw light upon the reason why the deplorable excess of carbon dioxide was present in so many rooms.

Before proceeding to the consideration of the amounts of air in circulation in our school rooms, it may not be out of place to recall briefly the facts and principles involved in the process of ventilation.

The process of breathing seems to be incessantly and continuously necessary for the maintenance of human life, and that for two reasons the individual must have about so many meals of oxygen to the minute, and is under the equally imperative necessity of exhaling such a volume of carbon dioxide and other respiratory excrements as to make the suspension of respiratory exhalation just as painful, dangerous and impossible as the suspension of inhalation. And further, respiratory carbon dioxide, and respiratory excrement, have the same chemistry outside of the lungs as within the lungs, and just as a man dies in a few moments if he may not breath out these poisons, so a room occupied by human beings becomes a place of discomfort, disease, and death or it breathes, inhales and exhales air, as incessantly and continuously as its human occupants, and in amounts determined by its size and their numbers.

This process of breathing a room, inhaling fresh air, and exhaling carbon dioxide and respiratory excrement, we call ventilation; and the amount of fresh air which the room must take in a minute a person, and the amount of foul air the room must extrude a minute a person, is determined by the average number of respirations of human beings a minute, the average cubic capacity of the human lungs and the toxic power of expired air. Scientific men have demonstrated that 3,000 cubic feet of air a person an hour is required in order that the respiratory food of a room may be sufficient to maintain comfort and health, and also in order that the toxic products of human respiration may not exceed the limit set by competent authority at 6 parts of carbon dioxide in 10,000 parts of air—the limit of permissible impurity.

Let us now see what the city chemist found to be the breathing capacity and breath record of the class rooms of our schools which breathe by the fan system. Attention is first arrested by the want of uniformity in the amounts of air received in the different rooms of the various schools and also in the different rooms of the same school. Experience has apparently taught our school builders that class rooms should be of about a given size, for they are generally found to have the same floor space, the same cubic contents and provide seats for the same number of pupils. Theoretically, rooms of the same cubic contents occupied by the same number of pupils and teachers should receive equal quantities of air, and have equal amounts of ventilation, but the facts disclosed by the city chemist demonstrated that inequality in air service at both inlets and outlets was the well nigh invariable rule. Air measurements at School No. 1, were made in 18 different rooms and the variation of the amounts inspired, the inlets was from 2158, the best room, to 1950, the next best, then to 1610, then to 1451, and so on down to the room having the poorer service of the school, with only 572, these several figures representing cubic feet of air a pupil an hour. At No. 18 of 17 rooms examined, the better rooms had respectively 2944, 2436, and 1800 and after this manner down to the bottom of the list, where we note 963 and 852 respectively.

This lack of uniformity appears in the work of expiration, the outlets, and is the rule of every school examined. Comment seems unnecessary.

Let us now observe the amounts of air entering the various rooms and see how they compare with the standard necessary for the preservation of health. This we take to be 3,000 cubic feet an hour a person and we find that in general and in particular the air supply is far below the standard. The better room of No. 1 has but .71 of standard, and the poorer room but .19. The average shortage of the 18 rooms of this school being .65. At No. 18 the better room shows .97 of standard and the poorer room .28. The average shortage of the 17 rooms of this school is .53. The above calculations relate to inspiration to inlet work only; when we look at expiration, outlet work, the showing is still worse. The better outlet at No. 1 gave .49 and the poorer outlet gave .16. At No. 18, the better outlet gave .61, and the poorer outlet gave .27. Taking the work at inlets and outlets we find in School No. 1, the fan ventilation ranges from .52 to .18 with an average for the school of .35. In No. 18, the fan ventilation ranges from .63 to .31, with an average of .47. The average for the two schools being .41. Again comment seems unnecessary.

A most important difference between theory and fact is to be noted in the nonrelation of inlet work to outlet work. The boast of the promoters of the fan system is its uniformity of performance under all conditions of wind and weather, and the theory is that the air which enters the inlet diffuses equally and universally throughout all parts of the room, warming, ventilating and furnishing breath to all the occupants in equal measure, and having done all this, then leaves the room by the outlets as foul air to make room for an equal volume of fresh air and this is incessant and continuous.

The inference is that the only force causing movement of air through the outlets is the force of the fan, and that there would be a constant relation between the amount of air entering by the inlet and leaving by the outlet and that the measure of the one would be the measure of the other. So generally has this fallacy been accepted even by men who should have known better that we find the city chemist taking it for granted, and doing his first winter's work without measuring or reporting upon the work of a single outlet. This important oversight having been corrected by the health department the work of the following year gives the required information.

The facts are, that the promises of the promoters of the fan system are not performed—that the amount of air going out at the outlet is not the same as that coming in by the inlet and practically speaking bears no relation to it. This conclusion is reached by tabulating the observations of the city chemist upon the various schools after the following manner.

The work of the inlets and outlets are placed in parallel columns from the higher to the lower. Theoretically, this should bring the inlets and outlets of the same room opposite or nearly opposite each other. That lines connecting the inlets and outlets of the same room should be horizontal or nearly horizontal.

The fact is that in 141 rooms of 7 schools, the lines are horizontal, as theoretically all should be, in 11 cases; the lines run down, as escape of air through crevices would permit, in 68 instances; and the lines run up from lower to higher in absolute defiance of the theory in 62 cases.

Comment upon these facts will be made in conclusion.

This seems to be the place to invite attention to the readiness of testing the work of the fan system of ventilation and air supply. In the vacuum system, the fan in the attic, the test has simply to show how much air is leaving the rooms by the outlets. It is plain that the ventilation can never be greater than the amount of air properly diffused would cause, and this should be at least 3,000 a person, an hour.

The efficiency of diffusion may be judged by the number and location of outlets, check upon the theory being made from time to time by observations upon the presence of carbon dioxide. The so-called plenum system requires no more complicated apparatus for its testing, but more measurements, there being at least one inlet and one outlet for each room and standard requirement gives the figure for a room at 6,000 cubic feet an hour a pupil. Here again it may be remarked, that the ventilation of a given room by the so-called plenum system can never be more than the sum of the work shown at the inlet and outlet, but may easily be less by reason of faulty location of the inlets and outlets. Any person able to tell the time of the day by the watch may use the anemometer and therewith make critical observations as to how near the work of the fan system approaches the standard. This seems of importance for the reason that the observations of the amount of carbon dioxide may only be accurately made by a practical and expert chemist; that under ordinary circumstances such observations require much time, complicated apparatus and considerable expense, whereas the air measurements require but a moment's use of a very simple instrument, the standardised anemometer.

Another interesting fact to be found in the city chemists reports is that of the considerable circulation into and out from the average class room after all possible pains have been taken to present such air movements. At No. 1, 5 inlets gave a volume of 12,053, while the outlets of these rooms showed only 6.002; at No. 16, 5 inlets gave a reading of 10,928, and the corresponding outlets, 5,609; at No. 18, the inlets of 5 rooms gave 15,892 and the outlets of these 8,569; at No. 19, the inlets of 5 rooms gave 9,774, and the outlets of these 4,131; at No. 56 the inlets of 5 rooms gave 10,733, and their outlets, 3,638. We find then as the measure of the porosity of the rooms of these schools and also as the nonrelation of inlets to outlets, that at No. 1, .49; at No. 16, .51; at No. 18, .53; at No. 19, .43; at No. 56, .33, only, of the inlet air reaches the outlets; and further that the work of the outlets is respectively .51, .49, .47 and.67 below the inlets.

Study of the tabulated work of the inlets and outlets of these schools gives the impression that the force acting upon and causing the air flow through the outlets is more uniform in its action, less liable to vagary than that governing the inlet flow. At No. 19 the five inlets having the lesser flow had a total of 5,565, and the outlet, of these rooms a total of 4,478. While a medium 5 inlets had a total of 8,522 and the outlets of these gave 3,664.

It will be seen by comparing these two groups of rooms in the same school, that while the inlet work rose 2,957, the outlet work fell off 814. At No. 56 the 5 inlets doing the better work gave a total of 11,095 and the outlets of these a total of 4,177; while 5 medium inlets gave a 'total of 9,412 and their outlets a total of 4,130, the difference in the inlets being 1,683 and of the outlets 47. The significance of this may appear later.

The study of the inlet and outlet work of the rooms found by the city chemist to have good ventilation furnishes food for thought. This is a total of 21 rooms, made up as follows: No. 1, 8 rooms; No. 12, 3 rooms; No. 16, 6 rooms; No. 18, 2 rooms, and No. 37, 2 rooms. In not one of these rooms is there standard inlet and outlet work and the average of the entire group shows, that even in case the fan should have credit for the circulation through both inlets and outlets, it would then be credited with only .47 of standard; the fact being that we are forced to conclude that in case it is necessary for perfect ventilation for 3,000 cubic feet of air a pupil, an hour, to enter and leave a room, then in the case of these rooms .53 of that air entered and left through other routes than the inlets and outlets.

It would seem that from this we are able to appreciate the significance and importance of the porosity—the accidental circulation of air through the class-rooms of our schools that are supposed to breathe by the fan system, and yet so deeply has the pseudo-scientific thought of the fan system corrupted the minds of our people, that a most intelligent medical man in discussing this question recently, seriously advised that rubber weather strips be applied to our windows in order to force the air to leave the room through the outlets.

During the last winter our city chemist and assistant bacteriologist visited the town of South Evanston, for the purpose of testing the air of a school said to have a simple and efficient system of ventilation.

This system, known in the trade as the Dickson system, is a gravity system that depends upon the law of the fluidity of air, that warm air is lighter than cold air, and in a flue or chimney will rise and continue to rise so long as the flue or chimney remains connected at its base with the outer cold air, and retains its power to warm that cold air. The Evanston school is built along the same general plan as our schools. The class rooms have about the same floor space and cubic contents and accommodate about the same number of pupils. The system of heating and ventilating is not very unlike the fan system, save that it is more simple. Steam coils are arranged in vertical flues, supplied with fresh air from below; the air thus warmed is delivered to the class rooms through inlet openings, one for each room, about eight feet above the floor.

The ventilator flues run in the same walls side by side with the inlet flues and are heated by them. The outlet flues are vertical, are chimney flues and run through the attic, through the roof and for some 8 or 10 feet above the roof. The inlet openings are of the same size for all of the rooms and are 25 x 28 inches. The outlet openings vary slightly in the different rooms and are 20 x 25 inches to 16 x 25 inches. The inlets and outlets are on the same wall surface, the outlet almost directly under the inlet and just above the floor.

A comparison of the air movements through the outlet flues of the Evanston and certain Buffalo Schools may be of interest. No. 45 of our city has the attic fan system and the sum of the outlet work of 5 rooms having the better records is 1,770. The outlets of 5 rooms having the poorer work gave 507. No. 1 of Buffalo has the fan in the basement. Its better 5 outlets give 6,194 and its poorer 5 outlets give 3,700. No. 18 Buffalo has the fan in the basement; its better 5 outlets give 7,488; and its poorer 5 outlets give 4,444. Evanston's better 5 outlets give 20,697; and its poorer 5 outlets give 14,226.

The Evanston system does not seem to be quite an ideal system. There seems to be doubts that a room of the dimensions of 24 x 32 feet or nearly that may be ventilated by a single outlet, and there is also chance for argument as to what the relation between the breathing line and the outlet opening, whether above or below or how much; but upon the question of taking air out of rooms through flues and that in standard quantities, the observations at Evanston indicate that gravity will do it.

Before concluding let us look for a moment for the reason why the out flues at Evanston seem so much more capable than those of Buffalo. And our first observation is that the reason is not from superiority of size, for those of Evanston have an area of 450 square inches, while those of No. 16 Buffalo have an area of 720 square inches. Neither is the advantage in location and direction, for in both cases the flues are in inside walls and are vertical and free from angles.

At No. 16 the average of 10 outlets, 5 better and 5 poorer, is 61,260 cubic feet an hour, sufficient to provide ventilation for 20 pupils. At Evanston, the average of 10 outlets, 5 better and 5 poorer, is 139,600 cubic feet an hour, enough for 46 pupils.

The flues at No. 16 were built under the fallacy that the plenum chamber would drive the air through them whether or no; they have a rough, careless, unfinished inside, they terminate in a cold attic, with no special connection with the outer air, only the general connection of a globe ventilator, and they have no special provision for keeping them warm, save as they are warmed by the air moving through them, whereby just so much levity is taken out of the air they are supposed to raise. Having no aid from the fan in the basement and not being expected to work by their own powers, having their natural powers seriously impaired by the cold attic, it is no wonder that their performance is less than half of their theoretical value, less than half those of Evanston, which although much smaller in opening were built to draw.

A careful consideration of the foregoing facts seems to invite the following conclusions:

1. The fan system of the Buffalo schools is a clumsy heating system, lacking in adaptability to different rooms and too liable to be either inadequate or wasteful. This is not of vital importance, as heating is only a matter of comfort and convenience.

2. The fan system of ventilation is a failure in inlet work, a more pronounced failure in outlet work, a failure in diffusion, a failure in displacement, a failure in the removal of air borne excrement, a failure in the removal of carbon dioxide; that it is not a plenum system, but a gravity system, there being no relation between the movement of air through the ventilators and the operation of the fan. That 141 different observations made in the rooms of 7 schools, showed not one instance of efficient ventilation as measured by motion of air at the outlets, and that in the tests of 196 different rooms of 9 schools, there was found 165 failures, as indicated by carbon-dioxide; and further, that in the rooms found to have adequate ventilation .53 of such ventilation was due to accidental causes—to the motion of air independent of either inlets or outlets. That this is a question of great importance inasmuch as the matter of ventilation of school rooms is not only one of comfort and convenience, but is essential to the preservation of health.

3. That the fan system is a demonstrated failure as an air supply, entirely lacking in uniformity and quantity and diffusion, as complete a failure in performance as it is absurd in principle, the surprise being that sane men would ever undertake to supplant and displace the simple method of natural air supply by anything so complicated and artificial as the fan system; remembering that to human beings air supply is at once a comfort and convenience and essential to health, and is also an instant, constant and immediate necessity to the maintenance of life.

4. That ideal school ventilation and air supply is a scientific question of absorbing interest, not alone because of the intricate questions of physics, chemistry, and physiology involved; but also in view of the fact that the tender, delicate and susceptible children of today, with their fearful liability to disease, liability to arrest of development of body, soul and spirit, are tomorrow the people.

That to our city the question is one of enormous financial and educational importance, because insufficient air supply and poor ventilation simply means, waste of effort and opportunity—teachers who may not teach and pupils who may not learn.

433 FRANKLIN STREET.

